# The optimal QTc selection in patients of acute myocardial infarction with poor perioperative prognosis

**DOI:** 10.1186/s12872-023-03594-0

**Published:** 2023-11-10

**Authors:** Xing Wei, Jun Feng, Zhipeng Zhang, Jing Wei, Ben Hu, Nv Long, Chunmiao Luo

**Affiliations:** 1 Department of Cardiology, The Second People’s Hospital of Hefei, Hefei Hospital, Anhui Medical University, Hefei, 230011 Anhui China; 2 The Fifth Clinical School of Medicine, Anhui Medical University, Hefei, 230032 Anhui China

**Keywords:** Acute Myocardial Infarction, AMI, QTc, MACCE, ECG, Correction formula

## Abstract

**Background:**

The predictive utility of QTc values, calculated through various correction formulas for the incidence of postoperative major adverse cardiovascular and cerebrovascular events (MACCE) in patients experiencing acute myocardial infarction (AMI), warrants further exploration. This study endeavors to ascertain the predictive accuracy of disparate QTc values for MACCE occurrences in patients with perioperative AMI.

**Methods:**

A retrospective cohort of three hundred fourteen AMI patients, comprising 81 instances of in-hospital MACCE and 233 controls, was assembled, with comprehensive collection of baseline demographic and clinical data. QTc values were derived employing the correction formulas of Bazett, Fridericia, Hodges, Ashman, Framingham, Schlamowitz, Dmitrienko, Rautaharju, and Sarma. Analytical methods encompassed comparative statistics, Spearman correlation analysis, binary logistic regression models, receiver operating characteristic (ROC) curves, and decision curve analysis (DCA).

**Results:**

QTc values were significantly elevated in the MACCE cohort compared to controls (P < 0.05). Spearman’s correlation analysis between heart rate and QTc revealed a modest positive correlation for the Sarma formula (QTcBaz) (ρ = 0.46, P < 0.001). Within the multifactorial binary logistic regression, each QTc variant emerged as an independent risk factor for MACCE, with the Sarma formula-derived QTc (QTcSar) presenting the highest hazard ratio (OR = 1.025). ROC curve analysis identified QTcSar with a threshold of 446 ms as yielding the superior predictive capacity (AUC = 0.734), demonstrating a sensitivity of 60.5% and a specificity of 82.8%. DCA indicated positive net benefits for QTcSar at high-risk thresholds ranging from 0 to 0.66 and 0.71–0.96, with QTcBaz, prevalent in clinical settings, showing positive net benefits at thresholds extending to 0-0.99.

**Conclusion:**

For perioperative AMI patients, QTcSar proves more advantageous in monitoring QTc intervals compared to alternative QT correction formulas, offering enhanced predictive prowess for subsequent MACCE incidents.

**Supplementary Information:**

The online version contains supplementary material available at 10.1186/s12872-023-03594-0.

## Introduction

Acute myocardial infarction (AMI) represents the most critical condition within the spectrum of acute coronary syndromes [1]. Diagnosis AMI relies on patient history, electrocardiogram (ECG) findings, and specific biomarkers alterations, including changes in cardiac troponin I (cTnl) and creatine kinase isoenzyme [2, 3]. The ECG’s capacity for risk stratification becomes particularly salient concerning the adverse prognoses of heart failure and all-cause mortality associated with AMI [4, 5].

ECG recording devices proficiently compute and display variations in the QRS and QT intervals [6]. Generally, the QT interval prolongs as the heart rate diminishes, necessitating a correction formula to adjust for heart rate dependency [7]. Studies indicate that heightened basal heart rate variability correlating with increased QTc variability augments the likelihood of QT correction formulas failing to negate the heart rate’s impact on QTc values [8]. An extended QTc interval, a hallmark of diverse cardiogenic electrophysiological disorders, often coincides with augmented dispersion of the ventricular refractory period [9]. Historical data has firmly established prolonged QTc interval as a pivotal contributor to ventricular fibrillation and ventricular tachycardia, directly implicating it in cardiac arrest scenarios prompted by torsade de pointes [10]. In addition, QTc holds substantial prognostic significance for microvascular complications in type 2 diabetes [11], atrial fibrillation [12], and mortality rates among heart failure patients [13]. Contrary to congenital long QT syndrome, the ventricular depolarization, as indicated by the ECG’s QRS complex in an ischemic condition, and ventricular repolarization, denoted by the QTc interval, trend to exhibit prolongation the short term. This phenomenon underpins the sustained alterations in QTc observed during acute myocardial infarction in patients [14, 15]. Furthermore, QTc interval elongation appears to precede ST-segment elevation amidst initial transmural ischemia in AMI patients [16].

Few studies have scrutinized the relative predictive capacities of assorted QT correction values for in-hospital MACCE in individuals with AMI. Opting for the most fitting QT correction formula is paramount for effective risk stratification, especially considering the pathological dynamics of AMI and the inherent instability of its prognostic factors.

## Methods and materials

### Study population and definitions

A total of three hundred fourteen patients (251 males and 63 females, average age 62.57 ± 13.74 years) who presented at our institution for emergency percutaneous coronary intervention (PCI) due to AMI between June 2018 and December 2022 were retrospectively assembled and examined. Baseline data encompassing age, gender, medical antecedents (diabetes, hypertension, old myocardial infarction, combined cerebral infarction), smoking status, Killip class, Gensini scores, and perioperative MACCE were meticulously accrued.

In-hospital MACCE was characterized as sudden cardiac death, shock, acute left heart failure, cerebral infarction, cerebral haemorrhage, and initial malignant arrhythmia within the hospital (including ventricular tachycardia and ventricular fibrillation, ventricular arrest, and third-degree atrioventricular block). Definitions for ST-segment elevation myocardial infarction (STEMI) and Non-ST-segment elevation myocardial infarction (NSTEMI) conformed to the pertinent guidelines [17, 18]. Specifically, STEMI was identified through elevated myocardial necrosis biomarkers (such as cTnI or creatine kinase isoenzyme) alongside ST-segment elevation of 1 mm or more in contiguous ECG leads, while NSTEMI was characterized by increased cTnI levels plus ischemic symptoms without ST-segment elevation. The exclusion criteria encompassed recent significant surgical procedures like percutaneous coronary angioplasty (< 3 months prior), pronounced renal insufficiency or liver dysfunction, a cancer diagnosis, contrast medium hypersensitivity, severe anemia, aortic coarctation, and the lack of clinical documentation.

This study complies with the Helsinki Declaration on Human Research. The need for study approval and informed consent was waived by the Ethics Committee of the Second People’s Hospital of Hefei.

### Grouping

Post screening via inclusion and exclusion parameters, three hundred fourteen AMI patients were recruited for this analysis. Participants were segregated based on the incidence of in-hospital MACCE events into two primary categories: (1) MACCE group (n = 81 cases), (2) Control group (n = 233 cases). Subsequent classification created three subdivisions based on in-hospital mortality: (1) Control group (n = 233), (2) MACCE_2_ group (non-fatal MACCE) (n = 67), and (3) MACCE_3_ group (in-hospital death group) (n = 14).

### Laboratory blood analysis and imaging examination

Blood laboratory indices encompassed white blood cells (WBC), platelets, K+, creatinine, high-density lipoprotein cholesterol (HDL-C), low-density lipoprotein cholesterol (LDL-C), total cholesterol (TC), triglycerides (TG), homocysteine, blood glucose, and cardiac troponin (cTnI) (drawn from a 5 ml sample of fasting venous blood the day following hospital admission). Imaging indicators included the left ventricular end-systolic internal diameter (LVs) and left ventricular end-diastolic internal diameter (LVd), left ventricular ejection fraction (LVEF), along with additional cardiac ultrasound and cardiac function parameters.

### Electrocardiogram (ECG) and QT interval correction formula

All AMI patients underwent 12-lead electrocardiogram examination before and after operation (electrocardiogram machine brand: FUKUDA DENSHI, specification model: FX-8222T). A 10-sECG sample was used for each ECG examination. QT interval was automatically measured by lead II ( threshold method ).The present study summarized and used Bazett [19]( QTcBaz = QT/RR^1/2^.

), Fridericia [20]( QTcFri = QT/RR^1/3^), Dmitrienko [21]( QTcDmi = QT/RR^0.413^), Framingham [22]( QTcFra = QT + 154 × (1 − RR), Schlamowitz [23]( QTcSch = QT + 205 × (1 − RR), Hodges [24]( QTcHod = QT + 1.75 × (HR − 60), Ashman [25]( QTcAsh = QT/log_10_(10 × (RR + 0.07)) × log_10_(10.7), Rautaharju [26]( QTcRau = QT + 242.51 − 434 × e^–0.0097 × HR^), and Sarma [27]( QTcSar = QT − 44.62 + 664 × e^–2.7 × RR^) for QT correction formula. The QTc derived from Bazett’s formula is referred to as QTcBaz, and the same applies to all other QTc. QTc is calculated from QT, HR, and RR (in ms).

### Statistical analysis

Data were analyzed statistically using SPSS 26.0 and R 4.2.3, and visualized through GraphPad Prism. Categorical variables are presented as percentages and compared using the chi-square test. Non-normally distributed variables are expressed as medians (P_25_, P_75_), and inter-group comparisons were conducted with the Mann-Whitney U test, while intra-group comparisons employed the Wilcoxon sign-rank test. Normally distributed indicators are articulated as mean ± standard deviation, with independent two-sample T-tests utilized for inter-group comparisons. The association between heart rate and QTc interval using different correction formulas was assessed through Spearman correlation analysis. Binary logistic regression models were constructed to determine whether different QTc values were independent risk factors for in-hospital MACCE events, and to compare the odds ratios (OR) between groups. Propensity score matching, based on regression analysis, was executed to contrast the QTc differences between groups, further mitigating confounding elements such as baseline demographics and laboratory data. The receiver operating characteristic (ROC) curve analysis was employed to ascertain the predictive value of each QTc interval for in-hospital MACCE events. Decision curve analysis (DCA) was utilized to appraise the clinical utility of diagnostic models. Patients experiencing in-hospital mortality were segregated further for statistical scrutiny of QTc intervals. Statistically significant with *P* value < 0.05.

## Results

### Comparison of baseline information and clinical data of MACCE group and control group

The study encompassed 314 AMI patients, of which 81 developed postoperative in-hospital MACCE. On average, the MACCE group was significantly older (*P* < 0.01) and presented higher Killip classification and Gensini scores compared to the control group (*P* < 0.01). STEMI patients exhibited a heightened probability of in-hospital MACCE events relative to control patients (*P* < 0.05) (Table 1). Significant disparities were noted between the groups concerning LVEF, LVs, WBC, K+, creatinine, glucose, homocysteine, CRP, and cTnI (*P* < 0.05). Conversely, there were no statistically significant differences regarding gender, past medical history, smoking history, LVd, QT interval, platelets, HDL-C, LDL-C, TG, and TC(*P* > 0.05) (Table 2).

**Table 1:** Analysis of population baseline information

Features	MACCE(n = 81)	Control group (n = 233)	X^2^/Z/T value	*P* Value
Female, n (%)	18 (22.2%)	45 (19.3%)	0.317	0.573
Age (years)	67.06 ± 13.608	61 ± 13.465	-3.460	0.001
Diabetes, n (%)	30 (37.0%)	66 (28.3%)	2.149	0.143
Hypertension, n (%)	52 (64.2%)	130 (55.8%)	1.742	0.187
History of infarction, n (%)	31 (38.3%)	106 (45.5%)	1.275	0.259
STEMI, n (%)	80 (98.8%)	214 (91.8%)	4.826	0.028
Smoking, n (%)	40 (49.4%)	126 (54.1%)	0.532	0.466
Killip class			65.498	< 0.001
Killip I, n (%)	31 (38.3%)	173 (74.2%)		
Killip II, n (%)	18 (22.2%)	49 (21.0%)		
Killip III, n (%)	12 (14.8%)	3 (1.3%)		
Killip IV, n (%)	20 (24.7%)	8 (3.4%)		
Gensini	81.00 (50.00, 101.50)	47.00 (35.00, 64.00)	-6.814	< 0.001
Intraoperative MACCE, n (%)	34 (41.98%)	47 (20.17%)	14.928	< 0.001

Normally distributed continuous variables are described by mean ± standard deviation, non-normally distributed continuous variables are quantified as median (interquartile range), and categorical variables are quantified as numbers (percentages)

STEMI, ST-segment elevation myocardial infarction; Killip class, clinical classification of heart failure; Gensini, coronary artery stenosis score; MACCE, Cardiovascular and cerebrovascular adverse events

**Table 2:** Comparison of laboratory indicators between MACCE group and control group

Features	MACCE(n = 81)	Control group (n = 233)	Z value	*P* Value
WBC (×10^9^/L)	11.06 (8.70, 14.10)	9.28 (7.29, 11.22)	-4.177	< 0.001
platelets (×10^9^/L)	192.00 (142.50, 236.50)	192.00 (157.00, 226.00)	-0.412	0.680
K^+^ (mmol/L)	3.81 (3.47, 4.13)	3.98 (3.70, 4.23)	-2.766	0.006
creatinine (µmol/L)	80.10 (68.00, 100.35)	70.40 (60.00, 84.20)	-3.151	0.002
TG (mmol/L)	1.39 (0.98, 1.98)	1.51 (1.08, 2.39)	-1.443	0.149
TC (mmol/L)	4.20 (3.57, 5.13)	4.29 (3.67, 4.97)	-0.182	0.856
HDL-C (mmol/L)	1.08 (0.95, 1.31)	1.06 (0.90, 1.20)	-1.167	0.243
LDL-C (mmol/L)	2.73 (2.08, 3.45)	2.73 (2.16, 3.32)	-0.129	0.897
blood glucose (mmol/L)	6.88 (5.81, 8.99)	6.00 (5.14, 7.61)	-3.304	0.001
Homocysteine (µmol/L)	17.70 (13.25, 20.97)	13.70 (11.05, 17.60)	-3.802	< 0.001
CRP (mg/dl)	23.08 (9.38, 42.63)	7.30 (3.32, 13.90)	-6.482	< 0.001
cTnI (µg/dl)	32.58 (9.21, 62.46)	7.82 (1.94, 42.7)	-4.092	< 0.001
LVEF (%)	55.00 (48.00, 60.00)	60 (56.00, 65.00)	-4.638	< 0.001
LVs (mm)	33.00 (30.00, 39.00)	32.00 (29.00, 35.00)	-2.852	0.004
LVd (mm)	49.00 (44.00, 53.00)	48.00 (44.00, 51.00)	-1.727	0.084
Preoperative				
QT (ms)	399.00 (356.50, 438.00)	392.00 (366.50, 417.50)	-0.585	0.558
QTcBaz (ms)	461.00 (430.00, 493.00)	432.00 (414.00, 451.00)	-5.974	< 0.001
QTcFri (ms)	436.03 (409.76, 471.40)	417.43 (402.15, 434.84)	-3.966	< 0.001
QTcDmi (ms)	445.72 (416.70, 478.44)	423.77 (408.78, 442.08)	-4.915	< 0.001
QTcAsh (ms)	450.38 (417.56, 479.49)	425.14 (408.93, 443.86)	-5.489	< 0.001
QTcFra (ms)	431.67 (403.95, 464.28)	415.95 (402.47, 432.63)	-3.247	0.001
QTcSch (ms)	443.51 (418.88, 476.01)	426.74 (410.88, 441.91)	-4.366	< 0.001
QTcHod (ms)	439.25 (418.63, 468.13)	417.00 (401.25, 433.75)	-5.514	< 0.001
QTcRau (ms)	441.58 (416.14, 471.52)	421.23 (407.63, 438.06)	-5.196	< 0.001
QTcSar (ms)	450.91 (418.91, 476.77)	423.33 (407.91, 440.85)	-6.283	< 0.001
Postoperative				
QT (ms)	381.00 (355.00, 413.00)	397.00 (373.50, 416.00)	-2.543	0.011
QTcBaz (ms)	443.00 (424.50, 462.00)	433.00 (410.50, 451.00)	-2.808	0.005
QTcFri (ms)	423.98 (401.22, 447.60)	419.34 (401.61, 436.13)	-1.234	0.217
QTcDmi (ms)	434.35 (412.74, 459.27)	425.01 (406.67, 443.26)	-2.405	0.016
QTcAsh (ms)	433.80 (415.80, 461.69)	425.31 (407.19, 444.17)	-2.721	0.006
QTcFra (ms)	422.51 (401.31, 443.67)	419.50 (402.89, 435.54)	-0.938	0.348
QTcSch (ms)	434.58 (417.24, 456.89)	428.00 (409.50, 444.85)	-2.415	0.016
QTcHod (ms)	423.25 (408.25, 444.50)	417.50 (400.88, 433.50)	-1.881	0.060
QTcRau (ms)	428.98 (412.45, 450.59)	422.42 (406.18, 438.85)	-2.351	0.019
QTcSar (ms)	430.90 (416.15, 454.46)	421.96 (406.61, 440.34)	-3.229	0.001

Normally distributed continuous variables are described by mean ± standard deviation, non-normally distributed continuous variables are quantified as median (interquartile range), and categorical variables are quantified as numbers (percentages)

WBC: white blood cells, TG: triglycerides, TC: total cholesterol, HDL-C: high-density lipoprotein cholesterol, LDL-C: low-density lipoprotein cholesterol, CRP: C-reactive protein, cTnI: cardiac troponin, LVEF: left ventricular ejection fraction, LVs: left ventricular end-systolic internal diameter, LVd: left ventricular end-diastolic internal diameter

### Preoperative and postoperative comparison of QTc interval in MACCE group and control group

**Fig. 1:**
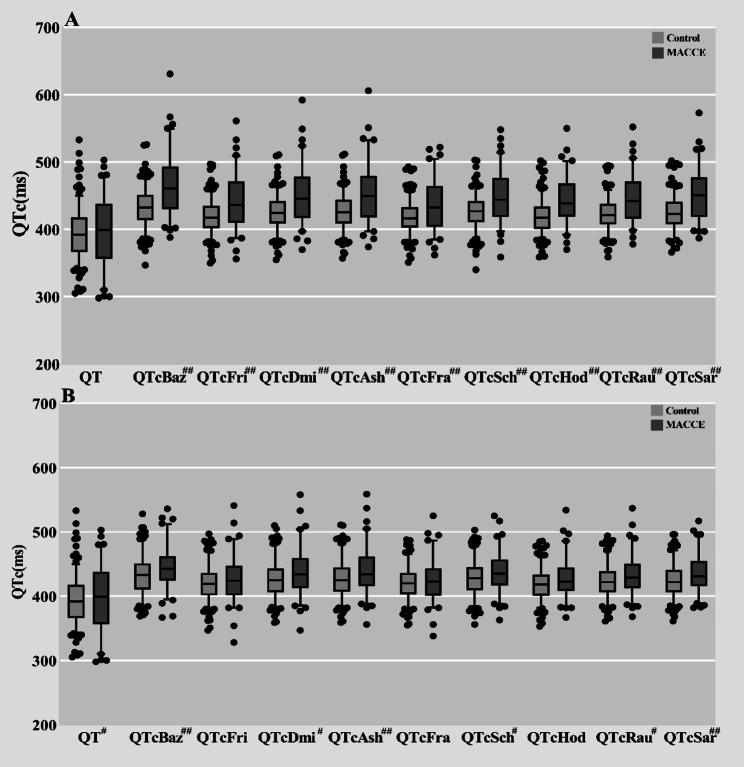
Comparison of QTc between MACCE group and control group. #: statistically different between the two groups, *P* < 0.05; ##: statistically significantly different between the two groups, *P* < 0.01. The comparison of QTc between the two groups is represented by the median (P25, P75). The median line represents the median distribution, the box range is from 25–75%, and the content of the error line is from 10–90%. Figure A: Comparison of QTc in patients with myocardial infarction before operation, there were statistical differences in QTc between the two groups (*P* < 0.05). Figure B: Comparison of QTc in patients with myocardial infarction after operation, there were significant differences in QTcBaz, QTcDmi, QTcAsh, QTcSch, QTcRau and QTcSar between the two groups (*P* < 0.05), but there was no significant difference in QTcFri, QTcFra and QTcHod between the two groups (*P* > 0.05)

The preoperative QT interval did not differ significantly between the two groups (*P* > 0.05). However, the values of each corrected QTc demonstrated statistical significance (*P* < 0.01) (Fig. 1A). Post-PCI, the QT interval, QTcBaz, QTcDmi, QTcAsh, QTcSch, QTcRau, and QTcSar were statistically distinct between the groups (*P* < 0.05), whereas QTcFri, QTcFra and QTcHod showed no significant differences (*P* > 0.05) (Fig. 1B).

### Comparison of the correlation between heart rate and different QTc intervals

**Fig. 2:**
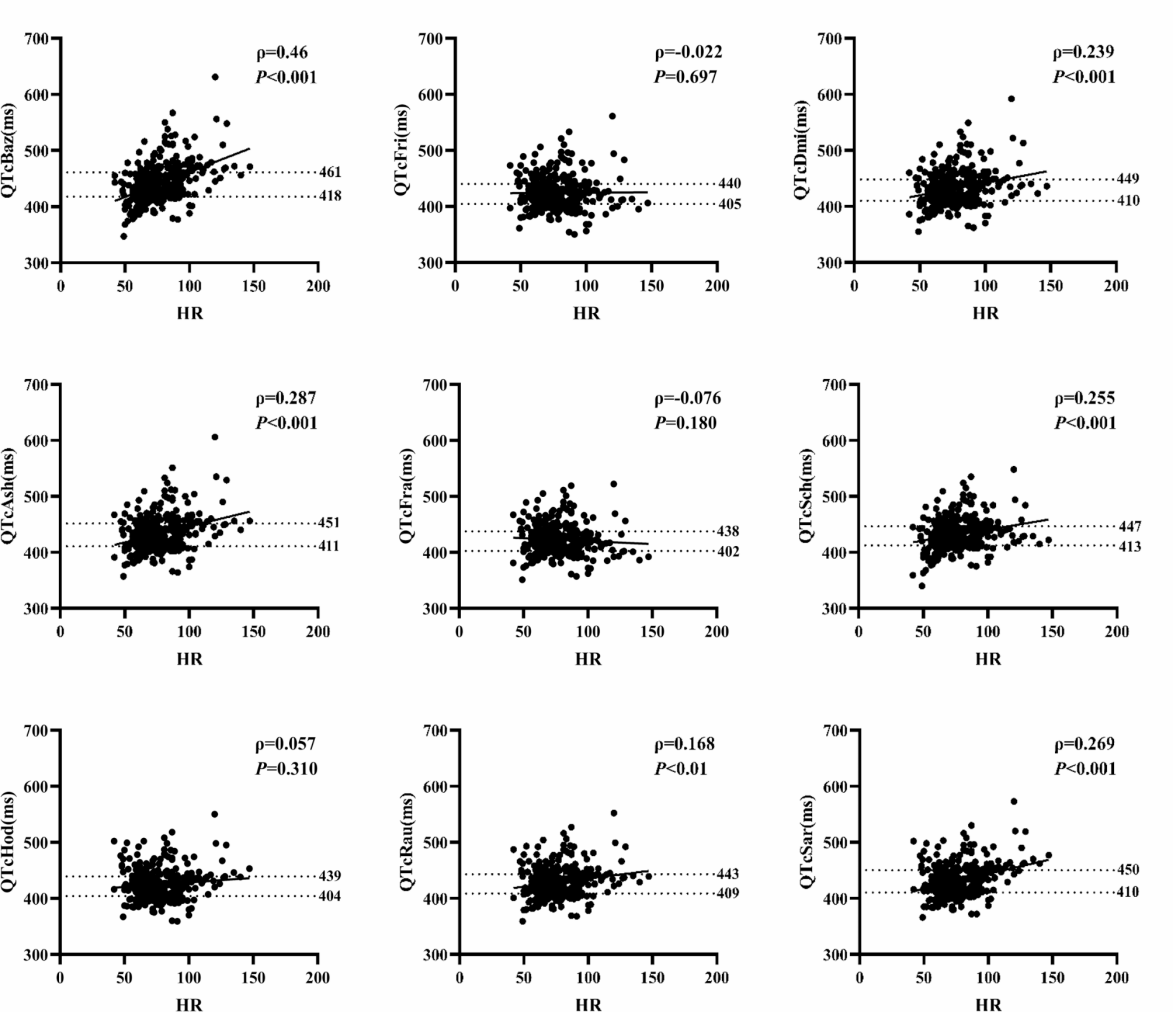
Correlation analysis of heart rate and QTc and the quartile interval of QTc. ρ: correlation coefficient

Spearman’s correlation analysis revealed positive correlations between heart rate and QTcBaz (ρ = 0.46, *P* < 0.001), QTcDmi (ρ = 0.239, *P* < 0.001), QTcAsh (ρ = 0.287, *P* < 0.001), QTcSch (ρ = 0.255, *P* < 0.001), QTcRau (ρ = 0.168, *P* < 0.01), QTcSar (ρ = 0.269, *P* < 0.001). Conversely, no significant correlations existed with QTcFri (ρ=-0.022, *P* > 0.05), QTcFra (ρ=-0.076, *P* > 0.05), QTcHod (ρ = 0.057, *P* > 0.05), and heart rate. The figure illustrates the corrected QT value [P25, P75] corresponding to changes in the ventricular rate (Fig. 2).

### Analysis of Independent risk factors for developing in-hospital MACCE in patients with AMI

This study employed univariate binary logistic regression analysis to determine if the QTc interval constitutes an independent risk factor for in-hospital MACCE in AMI patients (Table 3). Following multiple colinearity diagnostics, confounders, clinical data, and QTc interval were incorporated into a multifactorial binary logistic regression model. The findings indicated that the QTc interval, under various correction methods, emerged as an independent risk factor for in-hospital MACCE occurrences in AMI patients (*P* < 0.05). Notably, QTcSar had the highest hazard ratio (OR = 1.025), and QTcFra had the lowest hazard ratio (OR = 1.016) (Table 4).

**Table 3:** Single-factor binary logistic regression analysis of perioperative MACCE in AMI patients

Variables	B	SE	Waldχ [2]	OR	95%CI	*P* Value
Female	0.177	0.315	0.317	1.194	0.644–2.211	0.574
Age	0.033	0.010	11.304	1.034	1.014–1.054	0.001
Diabetes	0.398	0.272	2.135	1.488	0.873–2.537	0.144
Hypertension	0.351	0.267	1.734	1.421	0.842–2.396	0.188
History of infarction	-0.297	0.264	1.270	0.743	0.443–1.246	0.260
Smoking	-0.188	0.258	0.531	0.828	0.499–1.374	0.466
Intraoperative MACCE	1.052	0.278	14.304	2.863	1.660–4.938	< 0.001
Gensini	0.035	0.005	44.491	1.036	1.025–1.047	< 0.001
LVEF	-0.062	0.015	16.209	0.940	0.912–0.969	< 0.001
LVs	0.056	0.021	7.462	1.058	1.016–1.101	0.006
Killip class						
Killip (IIvsI)	0.718	0.338	4.520	2.050	1.058–3.973	0.033
Killip (IIIvsI)	3.106	0.674	21.211	22.323	5.953–83.700	< 0.001
Killip (IVvsI)	2.636	0.462	32.606	13.952	5.646–34.475	< 0.001
WBC	0.162	0.037	19.091	1.176	1.093–1.264	< 0.001
K^+^	-0.847	0.303	7.824	0.429	0.237–0.776	0.005
creatinine	0.018	0.006	8.201	1.018	1.006–1.030	0.004
blood glucose	0.090	0.034	7.053	1.094	1.024–1.169	0.008
Homocysteine	0.022	0.013	3.051	1.022	0.997–1.048	0.081
CRP	0.015	0.004	16.228	1.015	1.007–1.022	< 0.001
cTnl	0.009	0.003	9.448	1.009	1.003–1.015	0.002

B: regression coefficient, SE: standard error, OR: odds ratio, CI: confidence interval

MACCE: major adverse cardiovascular and cerebrovascular events, LVEF: Left ventricular ejection fraction, LVs: Left ventricular end-systolic internal diameter, WBC: white blood cell, CRP: C-reactive protein, cTnl: Cardiac troponin I

**Table 4:** Multifactorial logistic regression analysis of perioperative MACCE in AMI patients

	QTcBaz	QTcFri	QTcDmi	QTcAsh	QTcFra	QTcSch	QTcHod	QTcRau	QTcSar
**OR**	1.020	1.018	1.019	1.021	1.016	1.018	1.023	1.023	1.025
**95%CI**	1.008- 1.032	1.005- 1.031	1.007- 1.032	1.008- 1.034	1.002- 1.029	1.005- 1.032	1.009- 1.038	1.008- 1.037	1.011- 1.040
***P*** **Value**	0.001	0.008	0.003	0.001	0.021	0.008	0.002	0.002	< 0.001

OR: hazard ratio, CI: confidence interval

### Propensity score matching based on logistic regression analysis

To elucidate the differences in QTc between the two groups, this study utilized propensity score matching at a 1 : 1ratio, grounded in logistic regression analysis. Additional table S1 reveals that the baseline characteristics and biomarkers were effectively balanced across both cohorts. Subsequent to propensity score matching, the robustness of the QTc comparative outcomes persisted, with the QTcSar delineating the most substantial resilience in differentiation (*P* < 0.01).

### ROC curves for QTc interval predicting the occurrence of in-hospital MACCE in patients with AMI

**Fig. 3:**
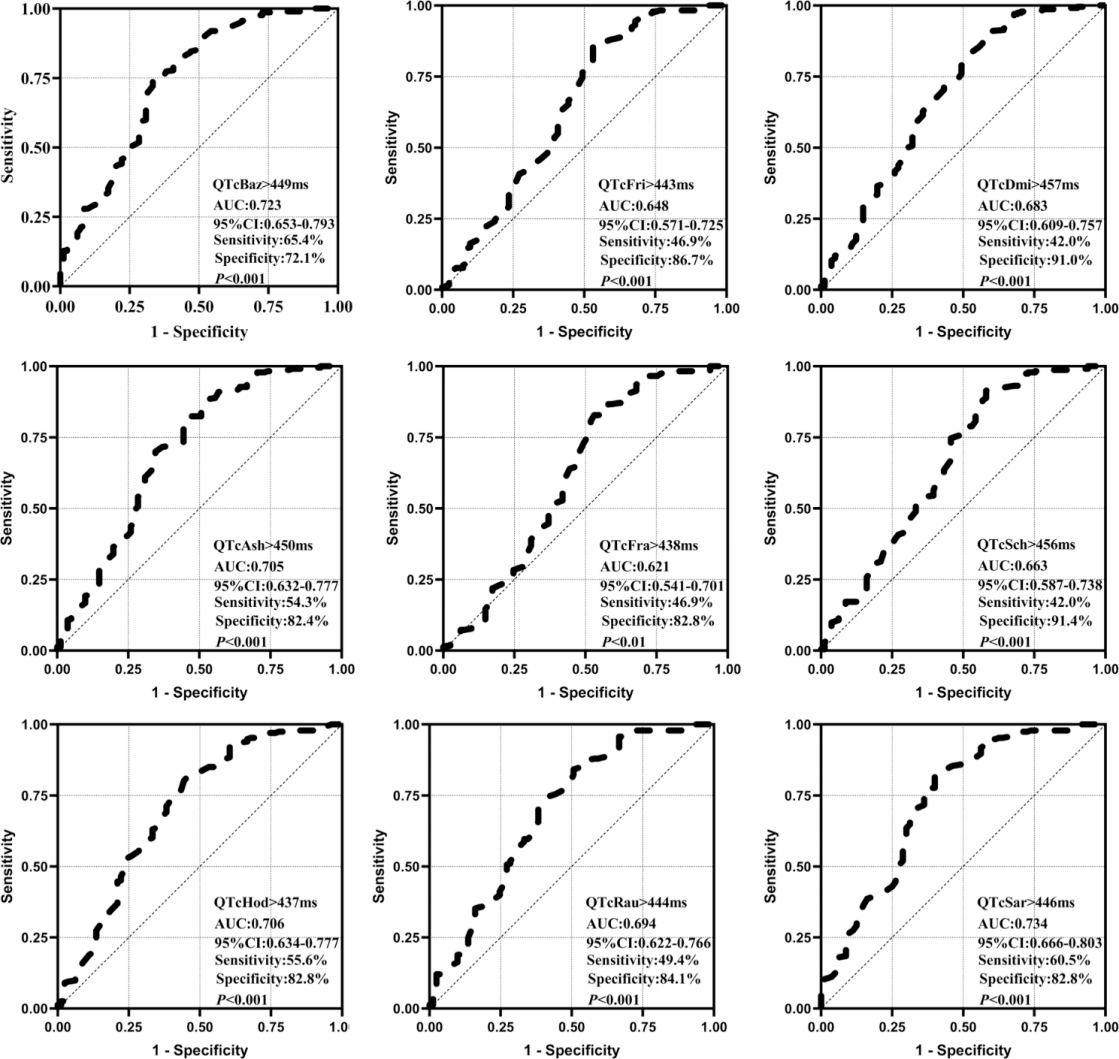
Predictive value of ROC curve analysis of QTc interval. AUC, area under the curve; CI, confidence interval; Sensitivity, sensitivity; Specificity, specificity

The ROC curves were instrumental in evaluating the QTc interval’s prognostic efficacy for in-hospital MACCE incidents among AMI subjects. QTcSar showed the best predictive value for MACCE in AMI patients (AUC = 0.734, 95% CI:0.666–0.803, sensitivity: 60.5%, specificity: 82.8%, *P* < 0.001) (Fig. 3).

### Decision curve analysis of QTcSar and QTcBaz

**Fig. 4:**
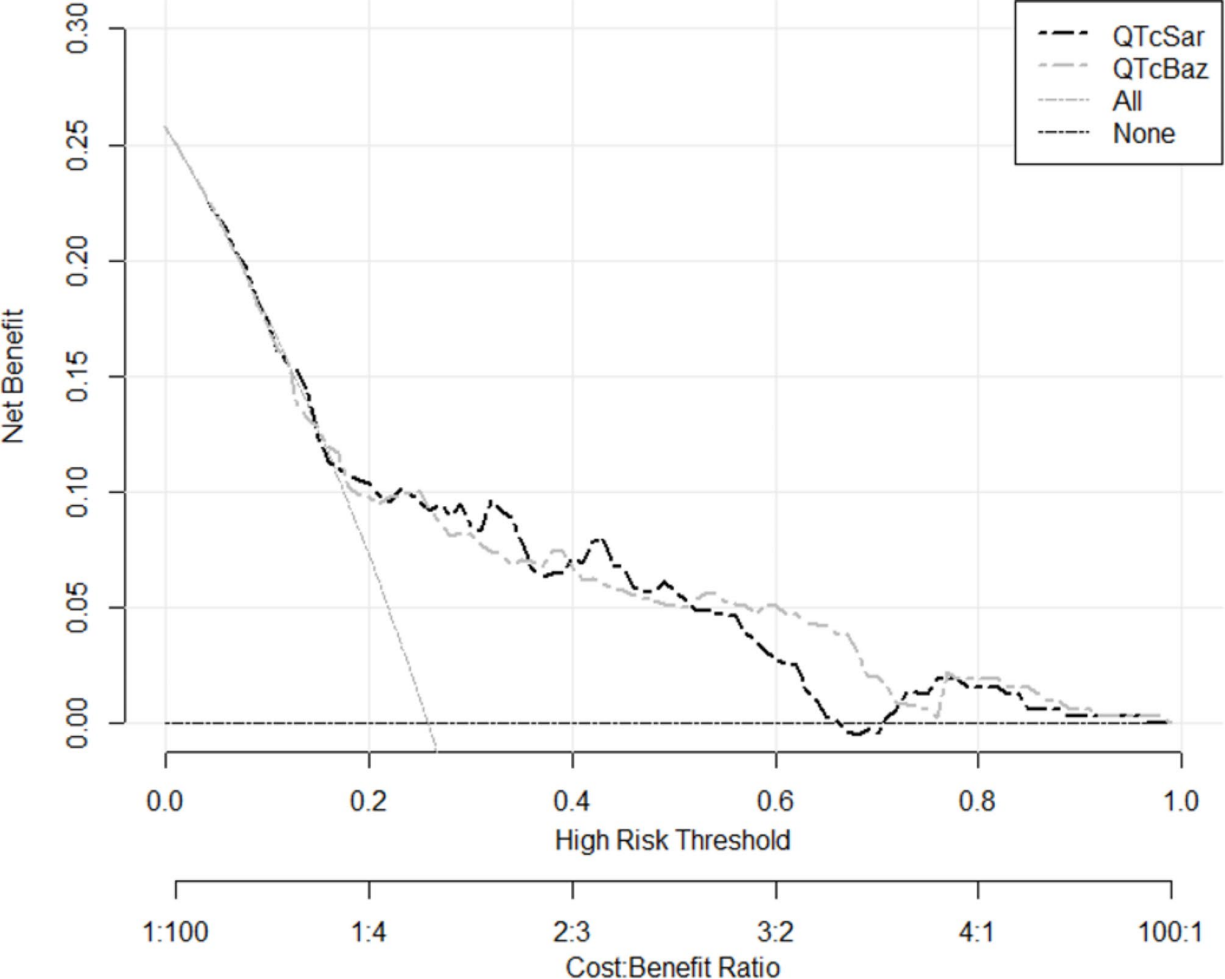
Decision curve analysis. Predictive models have significantly higher net returns than 0 when the risk threshold is 0.2–0.7. The net returns of QTcSar are higher than QTcBaz when the risk threshold is 0-0.51, and lower than QTcSar when the risk threshold is 0.51–0.72. This implies that the predictive model constructed on the basis of QTcSar is when the net returns are positive is more applicable

DCA curves indicated that QTcSar yielded a positive net benefit when the high-risk threshold ranged between 0 and 0.66 and 0.71–0.96, while QTcBaz demonstrated a positive net benefit within a 0-0.99 threshold. Furthermore, a reciprocal relationship was observed: the lower the high-risk threshold, the more pronounced the net benefit. Cumulatively, QTcSar surpassed QTcBaz in net benefit at thresholds spanning 0-0.51 but fell short within the 0.51–0.72 interval (Fig. 4).

### Statistical analysis of QTcSar of deceased patients

**Fig. 5:**
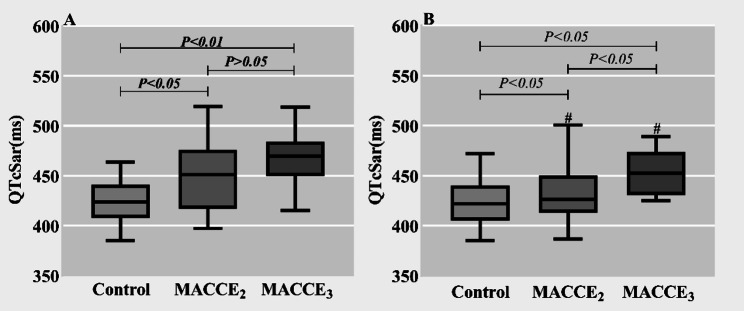
Inter-group and intra-group comparison of QTcSar in MACCE_3_ and MACCE_2_ groups and control groups. #: *P* < 0.05 compared with pre-intervention QTcSar in the same group

This study further examined preoperative and postoperative fluctuations in QTcSaramong in-hospital fatalities. Participants were segregated into control, MACCE_2_, and MACCE_3_ groups for comprehensive analysis. Pre-intervention, the QTcSar was markedly diminished in the control cohort relative to both MACCE groups (*P* < 0.05) (Fig. 5A); post-intervention, a significant divergence in QTcSar emerged across the triad (*P* < 0.05) (Fig. 5B). The results of the pre-and post-intervention comparisons within the same group showed a statistically significant decrease in QTcSar in the MACCE_2_ and MACCE_3_ groups after the intervention (P < 0.05). Interestingly, no statistical differences were identified in the changes in the control group pre- and post-intervention (*P* > 0.05) (Fig. 5).

## Discussion

This study established that AMI patients who manifested in-hospital MACCE exhibited consistently elevated QTc values relative to controls across diverse QT correction methods. Spearman’s correlation analysis between heart rate and QTc interval demonstrated an enhanced degree of variability in the frequently utilized QTcBaz, especially when heart rates exceeded 60 beats/min, consistent with the intrinsic variability of the baseline heart rate. After accounting for relevant confounders, each corrected QTc emerged as a distinct risk indicator for in-hospital MACCE among AMI patients (P < 0.05). Moreover, ROC curve analysis revealed that four types of QTc, encompassing QTcSar, QTcBaz, QTcHod, and QTcAsh, were markedly more predictive of in-hospital MACCE events among AMI patients than the other five QTc measures, with QTcSar standing out significantly (AUC = 0.734, 95% CI:0.666–0.803). Notably, the sub-group analysis of AMI patients demonstrated no significant changes in QTcSar within the control group (P > 0.05), whereas QTcSar experienced a significant reduction in the MACCE group (P < 0.05).

Alterations in QTc mirror both therapeutic interventions and repolarization anomalies, attributable to ion channel defects [10]. In the present study, emergency intervention effectively reduced the prolonged QTc interval in AMI patients. However, it was perplexing that the extended QTc interval, although improved postoperatively, was ineffective in decreasing the incidence of in-hospital MACCE.

In patients with cardiovascular disease (CVD), the QTc interval warrants substantial consideration [28]. A retrospective study substantiated that a prolonged QTc interval constitutes a risk factor for tip-twist ventricular tachycardia [29], with the association being even more pronounced amidst the prevalence of CVD [30]. Extensive research indicates a positive correlation between the QTc interval and the acute and/or long-term prognosis of conditions such as obstructive coronary artery disease [31], atrial fibrillation [12], and stroke [32] and can be used as a predictor of their risk. Previous studies have shown an association between QTc interval prolongation and increased mortality in patients with chronic heart failure (HF) [33, 34]. A comprehensive recent cohort study encompassing 1,800 HF subjects revealed that significant QTc prolongation (> 561 ms) correlated robustly with a heightened mortality risk among those experiencing acute heart failure, exhibiting a “U-shaped” relationship and a 30-day mortality rate of 9.7% [35]. Historical data indicate that the QTc interval tends to be substantially protracted in women relative to men, potentially due to variances in sex hormone secretion [36, 37]. In a cohort study featuring a 5-year follow-up period, investigators determined a potent association between QTc length and long-term mortality in male HF patients, subsequent to adjustments for confounders including age, body mass index, and medical history [38]. Various studies have shown that prolonged QTc is strongly associated with increased mortality in patients with acute and chronic heart failure. Previously, Bert Vandenberk [39] et al. have evaluated the different value efficacy of QT correction formulas such as Bazett, Fridericia, Framingham, Hodges, and Rautaharju on the risk of death during safety monitoring of drug use.

Furthermore, researchers concluded that for atrial fibrillation [40] and arrhythmias in advanced heart failure [41], the corresponding appropriate correction formula for QTc interval monitoring is of great clinical value in guiding medication use. In brief, selecting a proportionate QT correction formula is necessary for different CVD patients. The QTc interval during the acute period in AMI patients increases significantly based on autonomic nervous system disorders and myocardial electrical impulses due to cellular necrosis and electrolyte imbalance [42, 43]. Extended QTc interval has been demonstrated to predict mortality in survivors of myocardial infarction [15]. In ROC curve analysis, QTcBaz has a relatively high predictive value. However, we consider the quality of QTcBaz to be undesirable for evaluating the short-term prognosis of patients due to the potential instability of QTcBaz caused by the uncertain changes in heart rate in the acute phase of AMI patients. In contrast, the present study recommends the QTcSar derived from the Sarma correction formula as the QTc monitoring data for AMI patients during the perioperative period.

It is of concern that the QTc interval derived from each QT correction formula was an independent risk factor for postoperative MACCE in patients with AMI (*P* < 0.05). In analyzing risk factors among AMI patients experiencing MACCE events, a higher QTc value, as measured by the Sar formula, corresponded to an elevated risk of MACCE events (*P* < 0.001, OR = 1.025). Subsequent to propensity score matching grounded in logistic regression analysis, the comparison of QTcSar between groups proved more pronounced. Furthermore, this study determined that, relative to QTcBaz, adjusted via the Baz formula, QTcSar, modified by the Sar formula, exhibited reduced dispersion in conjunction with patients’ heart rates, indicating the Sar formula’s enhanced stability amidst diverse degrees of RR interval fluctuation. Our further assessment of QTc’s predictive capacity for AMI patients with MACCE events revealed that QTcSar possessed the superior predictive value. The nuanced detection of MACCE occurrences post-PCI in AMI patients holds critical importance; an extended QT interval, a precursor to potential malignant arrhythmias, demonstrates a stronger association between QTc and subsequent MACCE when adjusted using the Sar formula. Considering the significant variability in the dispersion between QTc and heart rate, as well as the correlation between QTc and MACCE, we assert that QTcSar holds greater applicability in the prognostic assessment of patients with AMI. However, the OR for each QTc interval exhibited variation. Consequently, it is imperative to factor in the rise in OR per unit increment in QTcSar interval when conducting postoperative risk stratification of patients, utilizing diverse QT correction formulas, to render more precise clinical determinations concerning treatment and the employment of pertinent medications.

### Limitations of the study

This study’s constraint lies in its design as a single-center retrospective study encompassing a limited number of cases. The presence of necrotic cardiomyocytes and autonomic dysregulation in AMI patients may engender unpredictable fluctuations in the preoperative QT interval, posing challenges to our data aggregation. Furthermore, the lack of systematic follow-ups post-discharge inhibited our ability to discern the incidence of long-term MACCE in postoperative patients.

## Conclusion

QTcSar has demonstrated superior predictive capacity for postoperative MACCE in AMI patients compared to QTc derived from alternative QT correction formulas. We endorse the use of QTcSar as a primary metric for monitoring QTc in AMI patients during the perioperative phase, thereby offering enhanced guidance for the pharmacological intervention in the postoperative setting.

### Electronic supplementary material

Below is the link to the electronic supplementary material.

Supplementary Material 1

## Data Availability

The data utilized in the current study can be obtained from the corresponding author upon reasonable request.

## Abbreviations

MACCE: Major adverse cardiovascular and cerebrovascular events
AMI: Acute myocardial infarction
ECG: Electrocardiogram
PCI: Percutaneous coronary intervention
STEMI: ST-segment elevation myocardial infarction
NSTEMI: Non-ST-segment elevation myocardial infarction
WBC: White blood cells
HDL-C: High-density lipoprotein cholesterol
LDL-C: Low-density lipoprotein cholesterol
TC: Total cholesterol
TG: Triglycerides
cTnl: Cardiac troponin I
LVEF: Left ventricular ejection fraction
LVs: Left ventricular end-systolic internal diameter
LVd: Left ventricular end-diastolic internal diameter
QTcBaz: QTc interval derived from Bazett’s formula
QTcFri: QTc interval derived from Fridericia’s formula
QTcDmi: QTc interval derived from Dmitrienko’s formula
QTcAsh: QTc interval derived from Ashman’s formula
QTcFra: QTc interval derived from Framingham’s formula
QTcSch: QTc interval derived from Schlamowitz’s formula
QTcHod: QTc interval derived from Hodges’s formula
QTcRau: QTc interval derived from Rautaharju’s formula
QTcSar: QTc interval derived from Sarma’s formula
OR: Hazard ratios
AUC: Area under the curve
CI: Confidence interval
ROC: Ratio of subjects working curves
DCA: Decision curve analysis
