# Supplementary material for: The optimal QTc selection in patients of acute myocardial infarction with poor perioperative prognosis
Source: BMC Cardiovasc Disord. 2023 Nov 10;23:551. doi: 10.1186/s12872-023-03594-0 (PMC10638740; doi:10.1186/s12872-023-03594-0)
Supplement: Supplementary file 1 — Supplementary Material 1 [file 12872_2023_3594_MOESM1_ESM.docx]

**Additional table S1** Propensity score matching based on logistic regression analysis

| **Features** | **MACCE(n=48)** | **Control group (n=48)** | **X^2^/Z/T value** | ***P* Value** |
| --- | --- | --- | --- | --- |
| Age (years) | 66.04±13.89 | 69.48±15.19 | 1.157 | 0.250 |
| WBC (×10^9^/L) | 9.66 (7.90, 12.36) | 9.99 (7.76, 11.29) | -0.088 | 0.930 |
| K^+^ (mmol/L) | 3.92 (3.52, 4.13) | 3.94 (3.60, 4.24) | -0.553 | 0.580 |
| creatinine (μmol/L) | 76.50 (67.00, 90.33) | 82.00 (69.75, 97.80) | 0.696 | 0.488 |
| blood glucose (mmol/L) | 6.51 (5.57, 8.45) | 6.28 (5.40, 7.66) | -1.033 | 0.302 |
| CRP (mg/dl) | 22.35 (7.58, 32.88) | 10.00 (4.82, 18.91) | -2.165 | 0.030 |
| cTnI (μg/dl) | 17.95 (6.71, 42.61) | 7.96 (1.83, 67.44) | -0.795 | 0.427 |
| Gensini | 58.00 (44.00, 88.25) | 59.00 (47.75, 82.50) | -0.062 | 0.950 |
| LVEF (%) | 56.00 (52.00, 60.00) | 58.00 (50.50, 61.00) | -0.602 | 0.547 |
| LVs (mm) | 33.00 (30.00, 39.00) | 34.00 (32.00, 37.25) | -0.411 | 0.681 |
| Intraoperative MACCE, n (%) | 12.00 (25.00%) | 13.00 (27.08%) | 0.054 | 0.816 |
| Killip class |  |  | 5.662 | 0.129 |
| Killip Ⅰ, n (%) | 25.00 (52.08%) | 26.00 (54.17%) |  |  |
| Killip Ⅱ, n (%) | 13.00 (27.08%) | 15.00 (31.25%) |  |  |
| Killip Ⅲ, n (%) | 7.00 (14.58%) | 1.00 (2.08%) |  |  |
| Killip Ⅳ, n (%) | 3.00 (6.25%) | 6.00 (12.50%) |  |  |
| QTcBaz (ms) | 458.00 (423.75, 490.25) | 439.00 (426.75, 451.25) | -2.286 | 0.022 |
| QTcFri (ms) | 441.00 (411.75, 467.50) | 424.00 (409.00, 434.25) | -2.089 | 0.037 |
| QTcDmi (ms) | 445.00 (415.00, 477.25) | 432.00 (421.00, 441.00) | -2.159 | 0.031 |
| QTcAsh (ms) | 449.50 (415.00, 478.25) | 432.00 (421.75, 442.25) | -2.404 | 0.016 |
| QTcFra (ms) | 435.50 (407.25, 465.25) | 424.00 (409.00, 434.00) | -1.766 | 0.077 |
| QTcSch (ms) | 444.00 (417.25, 474.00) | 432.00 (423.50, 442.25) | -1.763 | 0.078 |
| QTcHod (ms) | 439.50 (418.25, 467.00) | 425.00 (410.00, 431.50) | -2.759 | 0.006 |
| QTcRau (ms) | 445.00 (414.75, 466.75) | 428.50 (417.00, 435.25) | -2.455 | 0.014 |
| QTcSar (ms) | 450.50 (417.50, 469.50) | 431.00 (417.50, 437.00) | -2.877 | 0.004 |

Normally distributed continuous variables are described by mean ± standard deviation, non-normally distributed continuous variables are quantified as median (interquartile range), and categorical variables are quantified as numbers (percentages).
